# Whole-Genome Sequencing of an Isoniazid-Resistant Clinical Isolate of *Mycobacterium tuberculosis* Strain MtURU-002 from Uruguay

**DOI:** 10.1128/genomeA.00655-14

**Published:** 2014-07-17

**Authors:** Luisa Berná, Gregorio Iraola, Gonzalo Greif, Cecilia Coitinho, Carlos M. Rivas, Hugo Naya, Carlos Robello

**Affiliations:** a Unidad de Biología Molecular, Institut Pasteur de Montevideo, Montevideo, Uruguay; b Unidad de Bioinformática, Institut Pasteur de Montevideo, Montevideo, Uruguay; c Sección Genética Evolutiva, Facultad de Ciencias, Universidad de la República, Montevideo, Uruguay; d Comisión Honoraria de Lucha Antituberculosa y Enfermedades Prevalentes, Montevideo, Uruguay; e Departamento de Producción Animal y Pasturas, Facultad de Agronomía, Universidad de la República, Paysandú, Uruguay; f Departamento de Bioquímica, Facultad de Medicina, Universidad de la República, Montevideo, Uruguay

## Abstract

The incidence of tuberculosis in Uruguay has been effectively reduced to <30 per 100,000 population, although an increase in nonrisk populations in the last few years is evident. Here, we present the genome sequence of *Mycobacterium tuberculosis* strain MtURU-002 isolated from a patient showing bilateral pulmonary tuberculosis that was resistant to isoniazid.

## GENOME ANNOUNCEMENT

Tuberculosis (TB), caused by infection with *Mycobacterium tuberculosis*, constitutes a major cause of morbidity and mortality worldwide, ranking as the second leading cause of death from a single infectious agent, after human immunodeficiency virus. In Uruguay, the National Tuberculosis Program has effectively reduced the incidence of TB to <30 per 100,000 population, with 600 to 700 new cases per year (data available at the World Health Organization Web page [http://www.who.int/]). However, in the last 5 years, there has been an increase in TB incidence not only in high-risk populations (patients with human immunodeficiency virus and TB coinfection, those in poverty, and prisoners) but also in nonrisk populations, such as in our recent report of 11 cases of well-nourished young subjects affected by the disease (1). In this context, the whole-genome study of isolates from different populations (high- and low-risk) becomes a necessity in order to perform future comparative genomic studies and to determine new molecular markers of pathogenicity and transmissibility, among other aims. In this work, we performed the complete genome sequencing of a clinical isolate from a patient showing bilateral pulmonary tuberculosis that was resistant to isoniazid.

Sequencing was performed at the Institut Pasteur de Montevideo on an Illumina platform. A total of 1,496,856 paired-end reads (2 × 100 cycles) were generated; the reads were corrected using AllPaths-LG (2), and then Velvet (3) software was used for the *de novo* assembly. A total of 169 contigs were found, with an average coverage of 69-fold. Using the reference genome of *M. tuberculosis* H37Rv (accession no. NC_000962), the assembly quality was further improved through the PAGIT toolkit (4) and evaluated with the Assembly Likelihood Estimator (ALE) software (5). Finally, automatic annotation was performed using RAST (6). *M. tuberculosis* strain MtURU-002 has a total of 4,324,103 bp, with an average G+C content of 63%. It contains 4,328 predicted coding sequences (CDSs), 1 rRNA operon, and 45 tRNA genes. Single nucleotide polymorphisms versus *M. tuberculosis* H37Rv were identified using BWA (7) and the GATK pipeline (8). A total of 540 single nucleotide polymorphisms (SNPs) and 35 indels were found. Of them, 482 belong to coding sequences, and 8 introduce stop codons disrupting membrane protein-related genes involved in lipid metabolism or cell wall processes.

As mentioned, drug sensitivity analysis revealed the isolate to be resistant to isoniazid. Remarkably, we did not identify any reported mutation related to this resistance (https://tbdreamdb.ki.se). However, we found a novel mutation, G471S, in the *iniB* gene (isoniazid inducible gene) that might explain resistance. Further studies should be done to evaluate the ability of the *iniB* gene to confer isoniazid resistance

### Nucleotide sequence accession number.

This whole-genome shotgun project has been deposited at DDBJ/EMBL/GenBank under the accession no. JNGE00000000.
